# Nanosilver: Weighing the Risks and Benefits

**DOI:** 10.1289/ehp.121-a220

**Published:** 2013-07-01

**Authors:** Nate Seltenrich

**Affiliations:** **Nate Seltenrich** covers science and the environment from Oakland, CA. His work has appeared in *High Country News*, *Sierra*, *Earth Island Journal*, the *San Francisco Chronicle*, and other local and national publications.

It takes a special sort of case to spur attorneys into a debate over the drooling habits of toddlers. Yet that’s where lawyers from the Natural Resources Defense Council (NRDC), the U.S. Environmental Protection Agency (EPA), and Swiss chemicals company HeiQ found themselves in January 2013 as they debated in a federal appeals court the extent to which 1-year-olds and 3-year-olds chew, salivate, and swallow.^^1^^

At issue in the NRDC’s suit against the EPA, which is still awaiting ruling, was whether the agency was right in granting a conditional registration in December 2011 to a nanosilver-based antimicrobial fabric treatment manufactured by HeiQ.^^2^^ The EPA’s risk assessment was based in part on assumptions about exposure of 3-year-olds by sucking or chewing on nanosilver-laced textiles such as clothing, blankets, and pillowcases.

**Figure:**
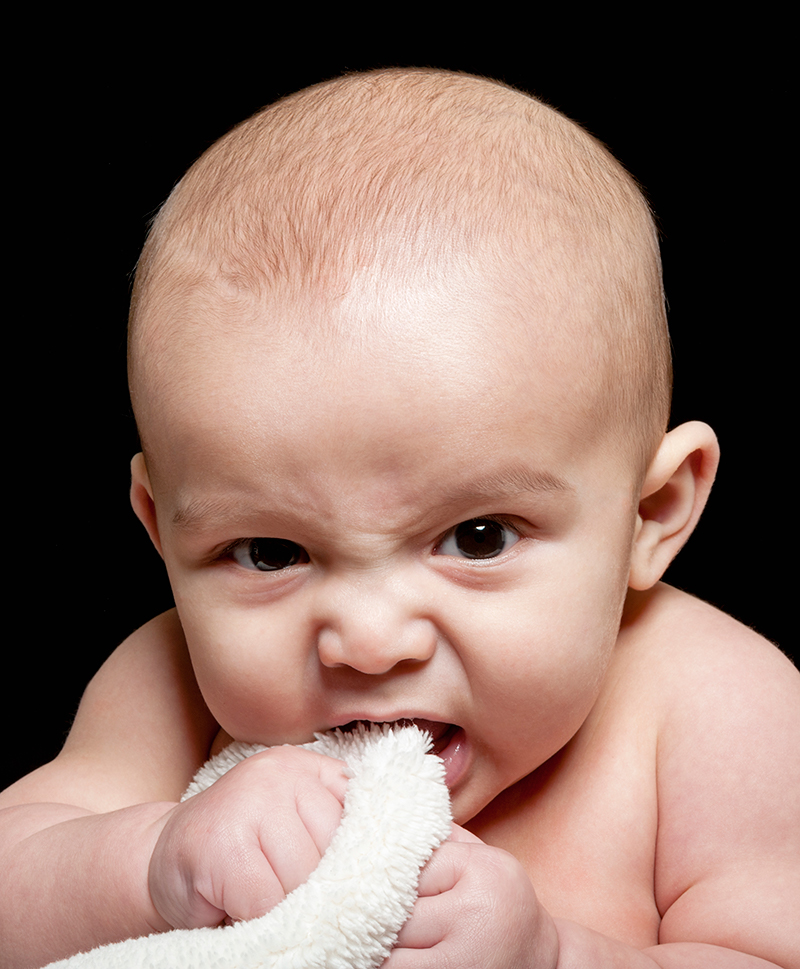
Silver nanoparticles are added to a variety of textiles and home goods as an antimicrobial. Although silver has been used safely for centuries, some question whether the rapid expansion of new exposure sources to nanosilver could have adverse consequences. © Tom Fullum/Getty Images

NRDC lawyer Catherine Rahm, however, begged to differ with the agency’s methods. In the January hearing, she argued that the agency record shows infants are more likely than any other subset of children to chew on fabrics that could contain the pesticide, and that if the agency were to recalculate its risk assessment based on the body weight of a 1-year-old, nanosilver concentrations in HeiQ’s product could result in potentially harmful exposures.

It’s an obscure but critical distinction as far as risk assessment goes. And given the implications for HeiQ and other companies looking to follow in its footsteps, the case has landed at the center of a prolonged conflict over the regulation of nanosilver and the growing deployment of this antimicrobial ingredient in a variety of commercial and consumer products.

Yet regardless of which side prevails in the case, the truth about nanosilver is not black and white. Even the loudest voices joining the NRDC’s call for strict regulation of nanosilver concede that context is key.

“I’m not somebody who is for or against nano,” says Ian Illuminato, who directs Friends of the Earth’s U.S. nanotechnology campaign, which has produced a series of reports on the potential dangers of nanosilver and other nanomaterials. “I’m just saying we need to do this in a mature and adult way. That’s not to say this isn’t a helpful technology and it doesn’t have a place in our future, but in the way it’s evolving right now, it’s very risky.”

Greg Lowry, a researcher at Carnegie Mellon University who studies the fate of nanoparticles in the environment, agrees that the use of nanosilver is a matter of balancing risk and reward. “There’s a tremendous potential benefit to the use in some of these products,” he says. “What one has to do is to ask yourself whether the benefits outweigh the risk.”

## Widespread Use

Nanosilver itself is nothing new. It has been used for different reasons in consumer and commercial products over the past century, although “nano” terminology does not always appear in the patent or scientific literature.^^3^^ Colloidal silver, in which silver particles down to the nanoscale are suspended in liquid, has been used for health and medical reasons since the early twentieth century and is now marketed as a dietary supplement and alternative medicine cure-all.^^4^^ Nanosilver has been used in the photo development process since the late 1800s and has been registered with the EPA for use in swimming-pool algaecides since 1954 and drinking-water filters since the 1970s.^^3^^

Recent advances in the ability to synthesize nanosilver particles have led to a surge of even more innovations.^^5^^ Most hinge on the ability to impregnate a wide range of materials and coatings with synthesized nanosilver compounds. “What we’ve learned how to do is bundle silver atoms into minuscule little particles, and then we’re able to take these particles and put them in places that they’ve never been able to get before,” explains Samuel Luoma, an emeritus researcher with the U.S. Geological Survey and author of *Silver Nanotechnologies and the Environment*, a report published by the Pew Project on Emerging Nanotechnologies.^^6^^

As a result, nanosilver has appeared in an increasingly wide range of products on U.S. shelves, among them electric shavers, athletic clothing, bed and bath linens, cosmetics, baby bottles, stuffed animals, keyboards, paints, and food containers. It’s also used in hospital equipment including catheters, stents, bandages, and wound dressings, as well as on surfaces including wheelchair seats and door handles. In Southeast Asia, nanosilver is used even more commonly and often openly; it has been sprayed in Hong Kong subways^^7^^ and touted on Korean toothpaste tubes,^^8^^ for example.

Despite its widespread use, nanosilver remains a fairly poorly understood material to both regulators and scientists. Consensus remains elusive on subjects as essential as how it behaves in the human body and the environment, and the extent to which its use may contribute to bacterial resistance.

Watchdog groups have seized on this murkiness in their continued call for tighter regulation of nanosilver. The NRDC, for example, points out that conditional registration of pesticides such as HeiQ’s product is based on the condition that complete toxicity data will be provided in a timely manner—meaning the product is allowed on the market while the company conducts the required studies.^^9^^ Meanwhile, the Silver Nanotechnology Working Group—an industry group formed to collect and disseminate data on nanosilver^^10^^—has argued ardently for its usefulness and safety, noting as well that most applications use very small quantities of the material.^^11^^

## A Powerful Antimicrobial

Recent evidence suggests that nanosilver’s form may not, in and of itself, be a factor in its germ-killing ability.^^12^^ But nanosilver does differ from macro forms of silver in a few key ways. First, the smaller form has a larger ratio of surface area to volume, which dramatically increases the potential for silver ions to be released—the primary mode of silver and nanosilver toxicity.^^13^^ In addition, nanosilver can go places in the body that larger silver particles can’t, and it may be small enough to enter cells^^14^^ or cross the blood–brain barrier.^^15^^ The real-world implications of this remain an area of ongoing research.

But with antibiotic-resistant infections a growing concern around the world, there’s a powerful incentive to develop new antimicrobial tools, and nanosilver offers great promise in this arena. A new nanosilver-based gel known as SilvrSTAT™, for example—introduced in fall 2012 as a wound-care antimicrobial by Utah-based ABL Medical—was proven in laboratory tests to kill strains of methicillin-resistant *Staphylococcus aureus* (MRSA) and vancomycin-resistant enterococci (VRE) within minutes, says ABL Medical managing director Keith Moeller. SilvrSTAT is also approved by the Food and Drug Administration (FDA) for use in elder-care centers to help treat diabetic and septic ulcers, surgical wounds, and grafted sites, Moeller says, and by the EPA for use as a disinfectant for hard nonporous surfaces.

**Figure:**
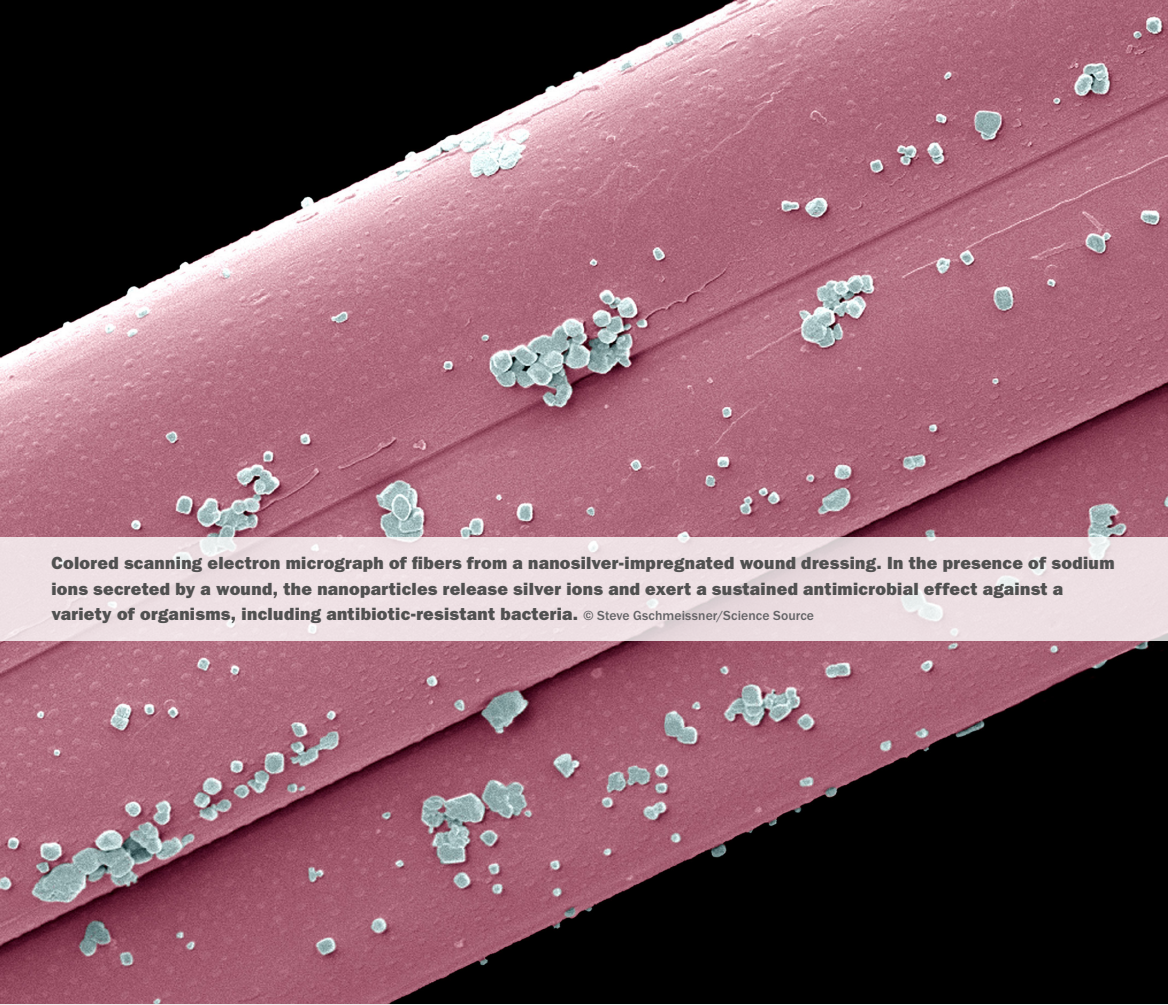
Colored scanning electron micrograph of fibers from a nanosilver-impregnated wound dressing. In the presence of sodium ions secreted by a wound, the nanoparticles release silver ions and exert a sustained antimicrobial effect against a variety of organisms, including antibiotic-resistant bacteria. © Steve Gschmeissner/Science Source

“We’ve never actually found a pathogen we couldn’t kill,” Moeller says. “That’s why silver is being used all over. It’s an incredibly safe, very broad-spectrum antimicrobial agent. It’s naturally occurring, and it’s just really effective.”

Nanosilver also plays a preventive role in special socks, shoes, and bandages developed for diabetics to prevent foot, ankle, and leg ulcers from progressing; in socks for military troops working in less-than-sanitary conditions to prevent trench foot, athlete’s foot, and other fungal infections; and in common athletic clothing to slow the spread of MSRA and other bacterial infections associated with close-contact sports.^^5^^

Rosalind Volpe, executive director of the Silver Nanotechnology Working Group, says nanosilver also deserves a place in common household goods. A paper by the working group explains, “Nanosilver antimicrobial treatments can bring a number of functionalities to consumer articles, including longer shelf life (e.g., cosmetics) giving more safety, less waste and ultimately lower prices for consumers; plastics that are protected against the degrading action of bacteria (e.g., discoloration); and textiles that are protected against colonization of bacteria that can lead to odors (e.g., sports clothing), ultimately giving greater comfort and prolonged use. Additional benefits such as reduced washing frequency at lower temperatures can give significant water and energy savings.”^^11^^

## How Much Is Too Much?

But the expansion of nanosilver into new applications may also contribute to unique risks. Looming large among them is the possibility that widespread use of nanosilver will contribute to silver resistance in bacteria, as has happened with other common antibiotics including penicillin,^^16^^ tetracycline,^^17^^ and triclosan.^^18^^

Nanosilver impregnated into consumer products and coatings will slowly, through laundering, be abraded from its substrate material at varying volumes and over varying periods of time, depending on concentrations in the material and the strength with which it is bonded, says Bernd Nowack of the Swiss Federal Laboratories for Materials Science and Technology. In consumer textiles, he notes, the industry standard is for the antimicrobial effect to persist over at least 50 washes, although some research suggests nanosilver can leach from certain products within the first few washes.^^19^^

Critics have questioned whether it’s wise to dispatch such a powerful weapon against bacteria in everyday contexts where bacteria pose a relatively minor concern. “It’s one thing if we’re using a little bit of nanosilver in the shoes of diabetics,” says Jaydee Hanson, policy director for the nonprofit International Center for Technology Assessment. “It’s another thing if you’re putting it in all underwear, all socks, every bed, every bed sheet. It’s a huge, exponential increase in the amount of nanosilver we’re putting into the environment.”

Gregory Crocetti, a Melbourne-based microbiologist who has worked on Friends of the Earth Australia’s nanosilver campaign, takes a stricter position. “Nanosilver should remain in a hospital setting only,” he says. “Those clinical uses will be diminished by completely hysterical and frivolous uses in homes. ... Nanosilver has a high likelihood of promoting not just silver resistance but also antibiotic resistance because of the process of co-selection.” Co-selection occurs when bacteria challenged with one antimicrobial find a resistance gene to it by swapping DNA with bacteria that are resistant to a different antimicrobial.^^20^^

## Environmental Considerations

Up to this point there has been limited documentation of silver resistance. These few cases largely have been isolated in *in vitro* studies rather than clinical or environmental settings.^^21^^^,^^^22^^^,^^^23^^^,^^^24^^ However, one 1975 article reported that silver-resistant *Salmonella typhimurium* had been isolated from a Massachusetts burn ward;^^25^^ the molecular basis for the resistance was proposed more than 20 years later.^^26^^

Some researchers and industry groups argue that silver and microbes have coexisted for billions of years and that resistance would have become evident by now if it were a viable threat. But Crocetti points out that silver was not historically used in the context of the widespread antibiotic resistance seen today.

“It is widely thought that different types of antimicrobial resistance genes have not been assembled by bacteria into such large collections on mobile genetic elements, particularly plasmids, until the last few decades,” Crocetti says. “It is important to note here that the *sil* operon—the major set of silver resistance genes—is regularly found alongside the cassettes of antibiotic resistance genes on many clinically important plasmids. This means that silver resistance has been regularly co-selected by, and can itself co-select for, other antibiotic resistance genes.”

Other research has shown that exposure to silver nanoparticles may, in some cases, improve bacterial survival rates. The authors of a widely cited study out of Rice University warned that low doses of silver may promote survival in what the authors called “an apparent hormetic effect.”^^12^^ Volpe agrees it may be prudent to develop and standardize silver minimum inhibitory concentration values and breakpoints, measures of how much of an agent is needed to effectively kill bacteria.

Studies have also investigated nanosilver’s toxicity to aquatic organisms^^27^^^,^^^28^^^,^^^29^^^,^^^30^^ and effects on human cells *in vitro.*^^31^^^,^^^32^^^,^^^33^^ Another area of interest—and considerable uncertainty—has been the behavior of the influx of silver nanoparticles into the environment, especially given elemental silver’s high toxicity to aquatic organisms, which is second only to mercury among metals.^^6^^ Potential impacts begin at the sewage treatment plants and septic tanks where nanoparticles end up after being washed down the drain. Their presence there raises at least two questions: Will they kill the bacteria needed to break down waste, and what happens when they find their way into soils and waterways when the sludge from sewage plants is applied to fields as fertilizer?^^34^^

Recent findings have suggested that some nanosilver particles are rapidly converted to more stable silver sulfides in certain oxygen-free environments where sulfates are present, such as wastewater plants. This significantly reduces the particles’ ability to release silver ions and kill bacteria^^35^^ (oxygen is required for the particles to release silver ions^^12^^). However, researchers still don’t fully understand how much total nanosilver is likely to be converted to silver sulfides, Lowry says, nor do they know the fate of either the nonsulfidized silver or the silver sulfides, which themselves are reactive with oxygen.

According to Luoma, environmental surveillance is a “critical requirement for a future risk management strategy.” Given the lack of methodologies that currently exist for routine monitoring of nanomaterials, he suggests that monitoring of silver itself in water, sediment, or biomonitors “could be a viable interim approach until methods specific to the nanomaterial are developed.”^^6^^

## Getting a Handle on the Numbers

Underlying this discussion so far is the assumption that what we’re doing actually matters—that the amount of silver, including nanosilver, that’s released into the environment and our bodies from consumer and hospital products is significant in relationship to background levels in the environment.

The Silver Nanotechnology Working Group isn’t so sure that’s the case. Nanosilver production has been estimated at 2.8–20 tons per year in the United States^^36^^ and 250–312 tons worldwide.^^37^^ Volpe says this represents a minor component of overall silver production—estimated by the silver industry to exceed 31,250 tons^^37^^—and of silver volumes in the environment.

Thus, Volpe says, new applications of nanosilver are not expected to have a significant impact on the level of silver demand over the next 10–15 years, even though many applications may achieve significant commercial success. The NRDC, in contrast, argues that “if nanosilver proves to be much more toxic than conventional silver, the smaller quantities released will not necessarily cause less harm.”^^9^^

Luoma noted in *Silver Nanotechnologies and the Environment* that silver is rare in the Earth’s crust and background concentrations extremely low. “Thus,” he wrote, “the addition of only a small mass of silver to a water body from human activities will result in proportionally large deviations from the natural conditions.”^^6^^ But he also pointed out that “the environmental chemistry of silver metal influences bioavailability and toxicity in complex ways.”^^6^^

Actual production data for nanosilver are not publicly available. Lowry believes the only way to get an accurate handle on how much nanosilver is entering the environment now and years down the line is to develop a comprehensive inventory of synthesis and production data. “With that kind of process, we can figure out what the loading on our waterways would be,” he says. “But without an inventory, it’s really hard to understand what our concentrations are. ... If you don’t know what the loading will be, you don’t know what the environmental concentrations will be.”
